# Synthesis of Tetrabenazine and Its Derivatives, Pursuing Efficiency and Selectivity

**DOI:** 10.3390/molecules25051175

**Published:** 2020-03-05

**Authors:** Seung-Mann Paek

**Affiliations:** College of Pharmacy and Research Institute of Pharmaceutical Sciences, Gyeongsang National University, Jinju Daero 501, Jinju, Gyeongnam 52828, Korea; million@gnu.ac.kr

**Keywords:** tetrabenazine, vesicular monoamine transporter type 2, Huntington’s disease, Parkinson’s disease, dopamine

## Abstract

Tetrabenazine is a US Food and Drug Administration (FDA)-approved drug that exhibits a dopamine depleting effect and is used for the treatment of chorea in Huntington’s disease. Mechanistically, tetrabenazine binds and inhibits vesicular monoamine transporter type 2, which is responsible for importing neurotransmitters from the cytosol to the vesicles in neuronal cells. This transportation contributes to the release of neurotransmitters inside the cell to the synaptic cleft, resulting in dopaminergic signal transmission. The highly potent inhibitory activity of tetrabenazine has led to its advanced applications and in-depth investigation of prodrug design and metabolite drug discovery. In addition, the synthesis of enantiomerically pure tetrabenazine has been pursued. After a series of research studies, tetrabenazine derivatives such as valbenazine and deutetrabenazine have been approved by the US FDA. In addition, radioisotopically labeled tetrabenazine permits the early diagnosis of Parkinson’s disease, which is difficult to treat during the later stages of this disease. These applications were made possible by the synthetic efforts aimed toward the efficient and asymmetric synthesis of tetrabenazine. In this review, various syntheses of tetrabenazine and its derivatives have been summarized.

## 1. Introduction

**Figure 1:**
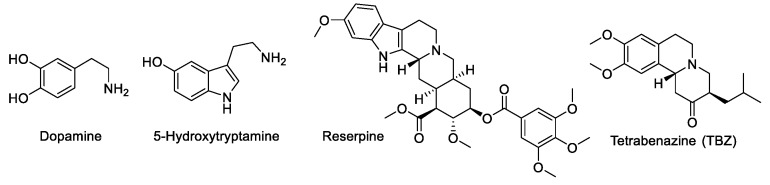
The structures of dopamine, 5-hydroxytryptamine, reserpine, and tetrabenazine (TBZ).

Dopamine, a catecholamine neurotransmitter, plays a pivotal role during signal transmission in the central nervous system (CNS). To release dopamine from the presynaptic neuron to the synaptic cleft, active transport of dopamine into the vesicle in neuronal cells occurs [1]. Vesicular monoamine transporter type 2 (VMAT2) is responsible for packaging dopamine in the neuronal vesicle by importing dopamine via an ATP-dependent mechanism [2]. Due to the importance of VMAT2 in controlling the dopamine levels found in the human body, numerous approaches to controlling VMAT2 have been studied toward the treatment of related diseases, such as Parkinson’s disease (PD) [3], Huntington’s disease (HD) [4], and schizophrenia [5]. Although the full understanding of and perfect cures for these diseases have not been achieved to date, early diagnosis or treatment of their symptoms have been possible after the development of various dopamine-based drugs [6]. Tetrabenazine (TBZ) is one of these drugs (Figure 1) [7,8,9,10].

**Figure 2:**
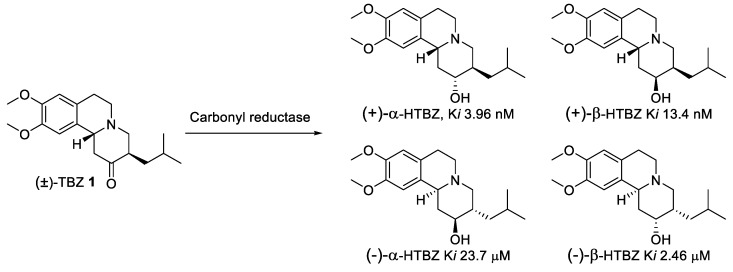
The metabolism of TBZ and the structures of its major metabolites.

TBZ **1** was introduced in 1957 [11]. Originally, TBZ was developed to change the levels of 5-hydroxytryptamine (5-HT) via the same mechanism observed for reserpine [11,12]. A mechanistic study on TBZ revealed it bound to VMAT2 in a highly selective manner (K*i* = 1.3 ± 0.1 nM), as expected [13]. Despite its original purpose as an antipsychotic drug, TBZ has been used to treat movement disorders such as chorea, tremor, hyperkinesia, akathisia, and tics in Britain since 1971 [14]. Based on these medicinal applications over the last 50 years, TBZ was approved for use to reduce chorea in HD by the US Food and Drug Administration (FDA) in 2008 [15]. In addition, the approval of TBZ has led to the further investigations on this classic molecule in terms of its stereoisomerism [16] and pharmacokinetics [17]. To overcome the stereoisomerism issue in particular, a variety of chiral separation [16,18] and asymmetric synthetic methods have been reported [19]. In addition to this problem, the rapid metabolism of TBZ is also a significant issue [17]. Earlier metabolomic studies have shown that the ketone moiety in TBZ is rapidly reduced by carbonyl reductase to afford α or β-dihydrotetrabenazine (HTBZ), as shown in Figure 2 [20]. In addition, a pharmacological study of these metabolites has also been carried out, which showed that α-HTBZ **2** is a more effective inhibitor of VMAT2 than its β-isomer [21]. More importantly, the binding affinities of these major metabolites were highly stereospecific. (+)-α-HTBZ binds to VMAT2 at a highly dilute concentration (K*i* 3.96 nM), whereas (–)-α-HTBZ did not bind to VMAT2 at the same concentration (K*i* 23.7 μM). It is not surprising that the further development of (+)-α-HTBZ for the treatment of movement disorders is still on-going.

**Figure 3:**
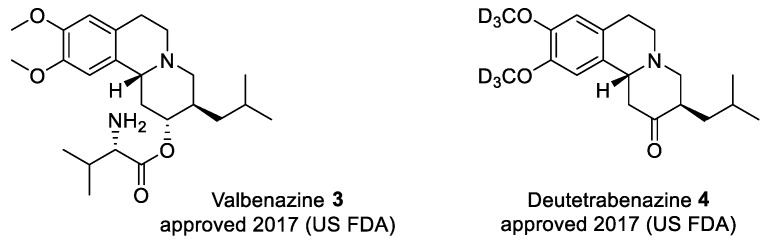
The structures of valbenazine and deutetrabenazine.

Valbenazine **3** is a prodrug of (+)-α-HTBZ. The (*L*)-valine ester group of the secondary alcohol in (+)-α-HTBZ increases the half-life of valbenazine (~20 h) [22] when compared to TBZ (~10 h) [23], supporting once-daily administration. Due to this improved pharmacokinetic profile, valbenazine was approved as a treatment for tardive dyskinesia by the US FDA in 2017 [24]. Deutetrabenazine **4**, the first US-FDA-approved deuterium-labeled drug, is also used to treat tardive dyskinesia [25]. This drug is a deuterium-labeled TBZ derivative that exhibits delayed demethylation and elimination, while the original mechanism of action of TBZ is unchanged. The introduction of deuterium leads to a more stable covalent bond and makes the molecule more stable against metabolism by enzymes such as cytochrome P450 (CYP) [26]. This metabolic stability gave rise to the development of a deuterium-substituted drug and the approval of deutetrabenazine in 2017. This drug allows less-frequent and low dosing, resulting in fewer side effects (Figure 3) [27].

**Figure 4:**
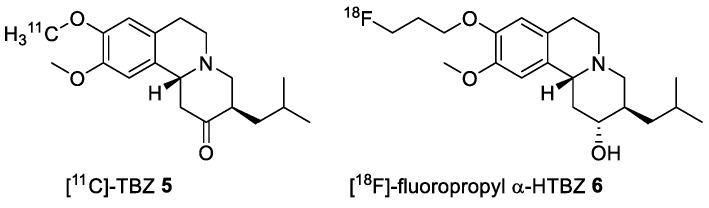
TBZ-based positron emission tomography (PET) imaging agents.

It is also noteworthy that the high affinity of TBZ toward VMAT2 led to the discovery of a powerful imaging agent used to identify the levels of VMAT2 in the brain [28]. Mechanistically, TBZ depletes dopamine and results in the improved treatment of movement disorders, such as chorea and tardive dyskinesia. However, this has also led to side effects, such as Parkinsonism and depression [29]. In PD patients, this highly selective and reversible VMAT2 inhibitor is disfavored due to these mechanistic and side-effect issues [30]. Attempts to utilize TBZ for the diagnosis of PD rather than as a therapeutic agent have been successfully carried out using radioisotopically labeled TBZ derivatives. Since the early 1990s, the introduction of ^11^C or ^18^F in TBZ has made it possible to measure the levels of VMAT2 using positron emission tomography (PET) [31,32]. The labile ^11^C or ^18^F isotope rapidly decays into ^11^B or ^18^O with the emission of a positron, which immediately collides with an electron. This collision creates a pair of γ-rays, which are detectable in PET. So far, tremendous studies of TBZ-based PET imaging agents, such as ^11^C-TBZ **5** or ^18^F-fluoropropyl-α-HTBZ **6**, have been reported (Figure 4) [33].

In terms of its structure, TBZ contains a benzoquinolizine skeleton with a 1,4-*anti* relationship between its alkyl substituents. This simple structure (MW 317) and highly important medicinal background has attracted significant interest from synthetic and medicinal chemists. Synthetic efforts have also driven the further development of TBZ-based drugs via positive feedback. Herein, the synthetic development of TBZ, including its racemic synthesis, chiral separation, and asymmetric synthesis, has been summarized.

## 2. Discussion

### 2.1. Racemic Synthesis of TBZ

**Scheme 1:**
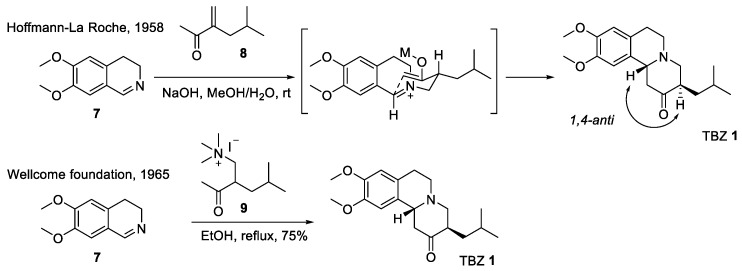
The classical synthesis of TBZ.

In 1958, Hoffmann-La Roche reported the synthesis of TBZ from dihydroisoquinoline **7** [34]. Treating **7** with enone **8** lead to a conjugate addition reaction, keto-enol tautomerization, and concomitant Mannich reaction to produce TBZ **1**. A chair-like transition state was used to explain the 1,4-*anti* relationship observed in the final product [35]. After this first synthesis, trimethylammonium salt **9**, a precursor of enone **8**, was successfully utilized under similar reaction conditions [36]. This simple procedure allowed the widespread use of TBZ for over 50 years (Scheme 1). It was also possible afterward to improve or modify this route for the synthesis of TBZ analogs by other pharmaceuticals, such as Cambridge Laboratories or Biovail [37].

**Scheme 2:**
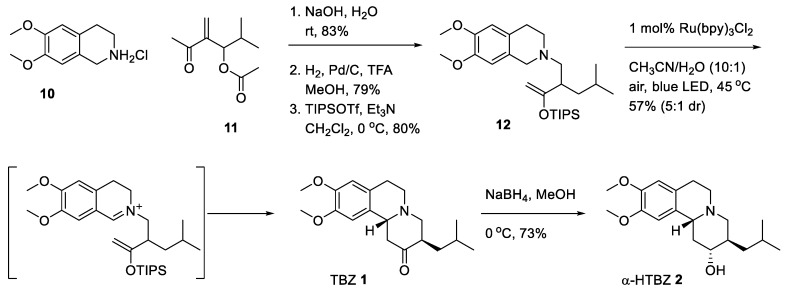
The oxidation–Mannich cyclization sequence used in the synthesis of TBZ. rt: Room temperature; TFA: Trifluoroacetic acid; TIPS: Triisopropylsilyl; bpy: 2,2’-bipyridine.

The use of a Mannich reaction, in situ oxidation using visible-light photo-redox catalysis, and a simultaneous cyclization strategy toward the synthesis of TBZ was reported in 2015, as shown in Scheme 2 [35]. This synthetic route features the use of tetrahydroisoquinoline **10** and an environmentally friendly photo-oxidation sequence [38]. Tetrahydroisoquinoline **10** was reacted with allyl acetate **11** to produce silyl enol ether **12** via a hydrogenation–silylation sequence. The benzylic *C-N* single bond was then oxidized to its corresponding iminium intermediate under light-induced oxidation conditions [39]. After extensive screening of the reaction conditions, the Marvin group optimized the reaction conditions using air, a ruthenium catalyst, and 8.5 W blue LED irradiation to produce TBZ **1** in moderate yield. TBZ **1** was subsequently reduced to give α-HTBZ **2**. 

**Scheme 3:**
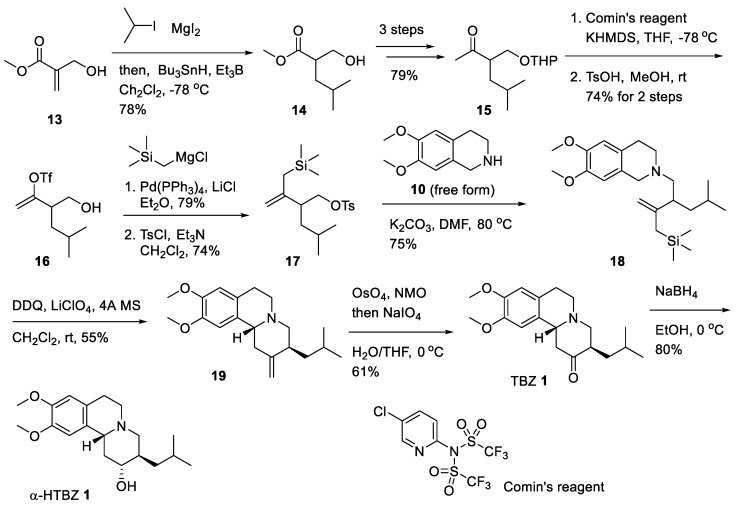
Synthesis of TBZ using an aza-Prins-type cyclization reaction. THP: Tetrahydropyran; HMDS: Hexamethydisilazane; THF: Tetrahydrofuran; Ts: *p*-toluenesulfonyl; Tf: Trifluoromethanesulfonyl; DMF: *N*,*N*-dimethylformamide; DDQ: 2,3-dichloro 5,6-dicyano 1,4-benzoquinone; MS: Molecular sieves; NMO: *N*-methylmorpholine *N*-oxide.

Scheme 3 shows an intramolecular aza-Prins-type cyclization reaction that has been utilized in the synthesis of TBZ. The Min group converted hydroxyl unsaturated ester **13** into primary tosylate **17** over eight steps [40]. The isopropyl group was first introduced to the conjugated alkene to furnish hydroxyester **14** in good yield. Ketone **15** could then be obtained in 79% yield over three steps using conventional functional group interconversion reactions, including protection, Weinreb amide formation, and methylation. Ketone **15** was resistant to conversion into the requisite allylsilane moiety using a Peterson-type olefination reaction [41]. However, **15** was converted into enol triflate **16** upon treatment with Comin’s reagent [42], followed by deprotection and the allylsilane moiety introduction via a Pd-catalyzed coupling reaction. **16** was reacted with an alkyl Grignard reagent in a Kumada cross-coupling reaction [43] to give the desired allyl trimethylsilane **17** after tosylation of the newly formed primary alcohol. The free amino group in **10** underwent a nucleophilic substitution reaction with the primary tosyl group in **17** to afford tertiary amine **18**. The pivotal in situ oxidation of the benzylic *C-N* single bond and resulting aza-Prins-type cyclization were then carried out [44]. Gratifyingly, the nucleophilic addition of the allyl trimethylsilyl group produced the desired benzoisoquinoline skeleton in moderate yield. Conventional alkene cleavage employing a dihydroxylation/diol cleavage reaction gave TBZ **1**. Reduction to give α-HTBZ **2** was also carried out using sodium borohydride.

### 2.2. Resolution of Racemic TBZ

Around 50 years of medicinal experience has proven TBZ to be safe when used as a racemic mixture for the treatment of chorea in HD or tardive dyskinesia. However, its side-effect profile, such as Parkinsonism and depression, still requires the preparation of enantiomerically enriched TBZ [7]. In addition, the highly stereospecific binding affinity of α-HTBZ to VMAT2 (K*i* 3.96 nM for (+)-α-HTBZ, K*i* 23.7 μM for (−)-α-HTBZ) demonstrates the importance of the asymmetric synthesis of TBZ [21]. However, the supply of enantiomerically pure material from the racemic synthesis shown above will not solve this issue. In this regard, the separation of (±)-TBZ using chiral HPLC has been studied. More interesting is that the chiral resolution of (±)-TBZ using an enzyme or chiral sulfonic/carboxylic acid leads to enantiomerically enriched TBZs or its diastereomeric salts, as shown below.

**Scheme 4:**
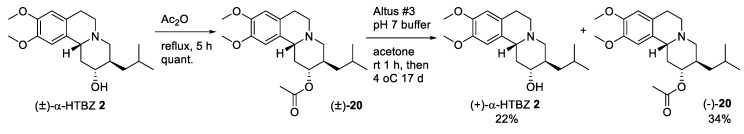
The chiral separation of racemic α-HTBZ using enzymatic resolution.

The first optically active stereoisomer of TBZs was reported by the Kilbourn group in 1997 (Scheme 4) [17]. In this report, simple acetylation and the following enzymatic hydrolysis were carried out to provide enantiomerically pure (+)-α-HTBZ and acetyl (−)-α-HTBZ in moderate yield (Scheme 4). During this procedure, 143 mg of (+)-α-HTBZ was prepared, as it was utilized to investigated a relationship of the stereoisomerism of TBZ and its binding affinity on VMAT2. Surprisingly, the (+)-enantiomer showed highly potent binding affinity, while the (−)-isomer did not bind it well. This pioneering research proved the importance of stereoselective preparation of (+)-TBZ or (+)-α-HTBZ in related drug development.

**Scheme 5:**
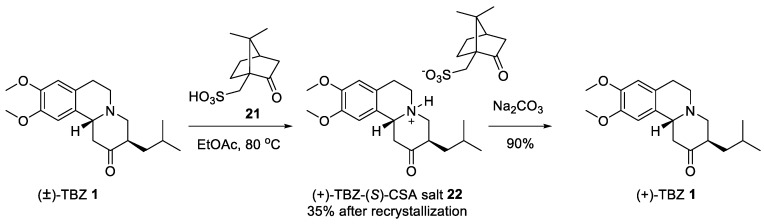
The chiral resolution of racemic TBZ using (1*S*)-(+)1−0-camphorsulfonic acid.

As it has been proven that chiral preparation of TBZ would be important, a more simple procedure for large-scale resolution of racemic TBZ has been studied. Actually, chiral acid was chosen to make diastereomeric TBZ salt that is easily separable. When racemic TBZ was treated with (1*S*)-(+)1−0-camphorsulfonic acid (CSA) **21** at 80 °C in a sealed tube for 28 h, the desired (+)-TBZ-(+)-CSA salt **22** could be selectively recrystallized after filtration to remove the excess (+)-CSA and additional storage at room temperature. Single X-ray crystallography and continuous measurement of the optical rotation proved its absolute configuration and enantiomeric purity. Using this simple resolution protocol, the Greig group executed a simple basification step to afford (+)-TBZ in its free amine form (Scheme 5) [16].

**Scheme 6:**
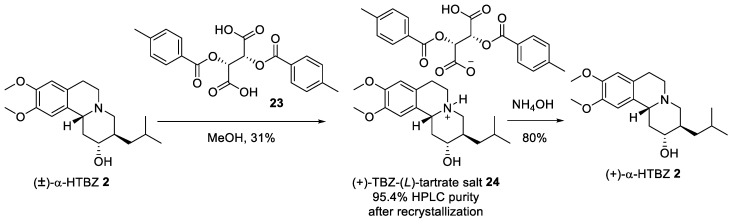
The chiral resolution of racemic α-HTBZ using a tartaric acid derivative.

The racemic mixture of α–HTBZ can be also separated using a similar protocol, as shown in Scheme 6 [18]. *p*-Toluoyl-(*L*)-tartaric acid **23** was used instead of (+)-CSA **21** to convert racemic α–HTBZ into its separable (+)-α–HTBZ-(*L*)-tartrate salt **24** after recrystallization (31% yield, 95.4% HPLC purity). Successive recrystallization increased the purity of the salt up to 100% by HPLC. The salt was converted into (+)-α–HTBZ **2** upon treatment with aqueous NH_4_OH. Impressively, this resolution was carried out on a large scale (>20 g). (–)-α–HTBZ could also be separated from the supernatant obtained from the recrystallization process using *p*-toluoyl-(*D*)-tartaric acid.

### 2.3. Asymmetric Synthesis of (+)-TBZ

Asymmetric synthesis is another solution for the preparation of enantiomerically enriched (+)-TBZ. For this purpose, asymmetric synthetic routes toward (+)-TBZ and (+)-α–HTBZ have been developed. Upon the approval of racemic TBZ by the US FDA in 2008, some effective synthetic strategies have been reported, as outlined below.

**Scheme 7:**
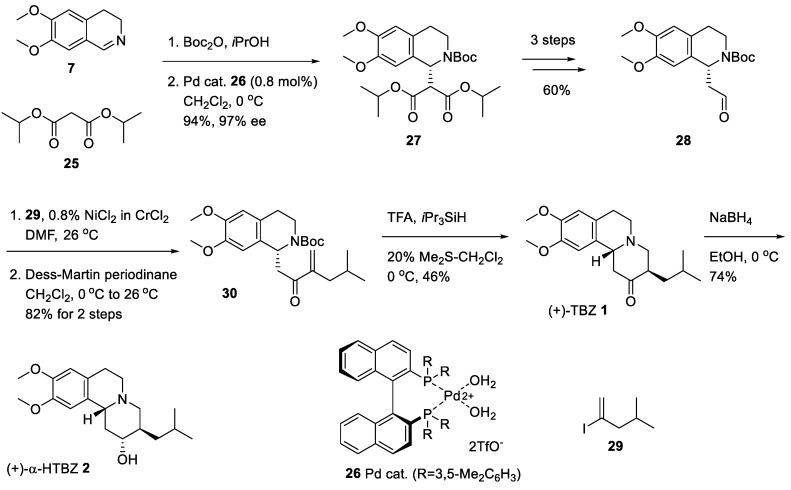
Asymmetric synthesis of (+)-TBZ and (+)-α-HTBZ using an asymmetric malonate alkylation reaction. Boc: *t*-butoxycarbonyl; ee: Enantiomeric excess.

Scheme 7 shows the first asymmetric synthesis of (+)-TBZ [19]. The crucial chiral center in TBZ was introduced at a very early stage of the synthesis. After activation of free imine **7** to its corresponding iminium ion using *t*-butoxycarbamate, a Sodeoka Pd-catalyzed asymmetric malonate alkylation reaction was carried out [45]. A survey of the Pd catalyst and various malonate esters showed that the Pd catalyst **26** and diisopropylmalonate **25** were suitable for producing the desired product **27** in 94% yield and 97% ee. β-Amino malonate **27** was then transformed into aldehyde **28** via a three-step protocol, including hydrolysis, decarboxylation, and semi-reduction, upon careful treatment with hydride. Interestingly, the direct Krapcho decarboxylation reaction of **27** produced a complex mixture of side products [46]. Another C-C bond forming reaction to introduce the remaining carbon framework of TBZ was then carried out by converting aldehyde **28** into unsaturated ketone **30,** followed by the addition of a vinyl anion and oxidation of the resulting alcohol. When vinyl iodide **29** was treated with NiCl_2_ in CrCl_2_, the desired Nozaki–Hiyama–Kishi reaction [47] occurred to produce an intermediate allylic alcohol, which was oxidized to ketone **30** using the Dess–Martin periodinane [48] in an overall 82% yield. Finally, Boc deprotection under acidic conditions induced the desired *6-endo-trig* cyclization reaction [49] to occur via a conjugate addition reaction between the newly formed amine and the unsaturated ketone. Employing this cyclization reaction, (+)-TBZ could be obtained with a 46% yield as a separable 5:1 mixture of diastereomers. (+)-α-HTBZ could also be produced as a separable 5:1 mixture of diastereoisomers using sodium borohydride. 

This was the first asymmetric synthesis of (+)-TBZ performed without using chiral resolution or chiral HPLC. This strategy opened up a new chapter for the development of other asymmetric syntheses of (+)-TBZ and (+)-α-HTBZ.

**Scheme 8:**
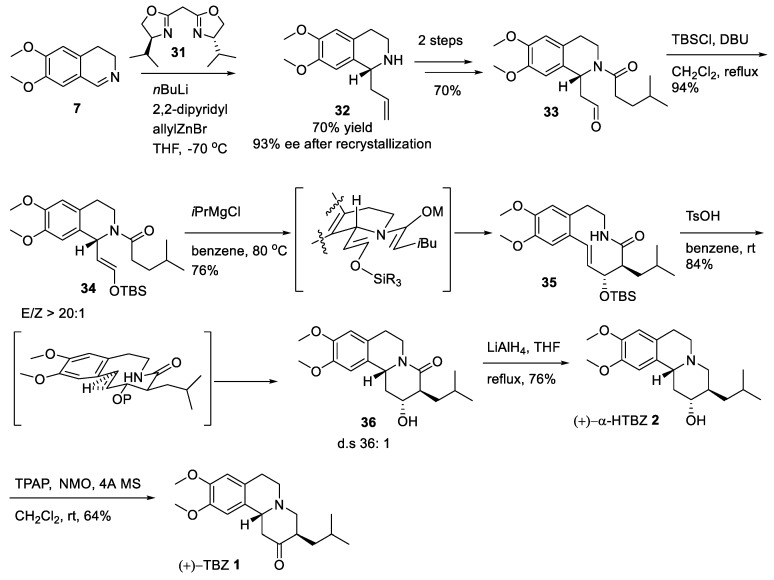
Asymmetric synthesis of (+)-TBZ and (+)-α-HTBZ using an aza-Claisen rearrangement reaction. TBS: *t*-butyldimethylsilyl; DBU: 1,8-diazabicyclo[5.4.0]undec-7-ene; TPAP: Tetrapropylammonium perruthenate.

The Suh group reported the second asymmetric synthesis of (+)-TBZ in 2010, as shown in Scheme 8 [50]. This synthetic route features a selective enol etherification and its use in an aza-Claisen rearrangement reaction. A Nakamura asymmetric allylation [51] was performed to create the first chiral center in the isoquinoline skeleton. Employing bisoxazoline ligand **31** and freshly generated allylZnBr, the desired allyl group was added to imine **7** to produce secondary amine **32** in 70% yield with moderate enantioselectivity. After isovaleryl aldehyde **33** was obtained over two conventional steps involving the formation of the amide using isovaleric acid and dihydroxylation/diol cleavage, it was transformed into *trans*-silyl enol ether **34** in the presence of a strong amine base and TBSCl in refluxing CH_2_Cl_2_ [52]. Gratifyingly, the *trans* geometry of the resulting alkene moiety could be produced with excellent selectivity (*E*/*Z* >20:1). 

The pivotal aza-Claisen rearrangement of enol ether **34** was then carried out [53]. The use of LHMDS (Lithium bis(trimethylsilyl)amide) in refluxing toluene afforded the desired 10-membered lactam **35** in 25% yield, but surprisingly, the Grignard reagent resulted in an improved deprotonation and concomitant aza-Claisen rearrangement reaction, which gave product **35** in 78% yield. Further studies revealed this superior result originates from the steric interaction between the alkoxide-cation complex and bulky isobutyl side chain [54]. Based on this finding, more examples showed the Grignard reagent could be used as an advanced base for the aza-Claisen rearrangement. With the desired macrolactam **35** in hand, a transannulation reaction under acidic conditions was carried out to construct the benzoquinolizidinone skeleton of TBZ [55]. The high diastereoselectivity observed in this reaction is thought to be a result of the chair-like transition state. Due to the acidity of the reaction conditions, the labile silyl ether group was also cleaved in the reaction. Finally, LiAlH_4_ reduction of amide **36** afforded (+)-α-HTBZ **2** uneventfully. Ruthenium-catalyzed oxidation of the secondary alcohol in (+)-α-HTBZ produced (+)-TBZ **1** in moderate yield.

**Scheme 9:**
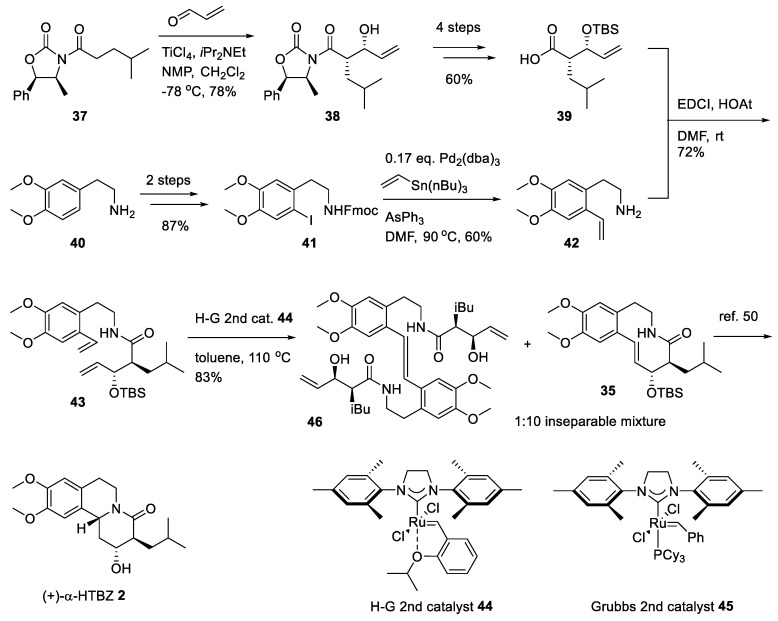
Asymmetric synthesis of (+)-α-HTBZ using ring-closing metathesis. NMP: *N*-methylpyrrolidine; Fmoc: Fluorenylmethoxycarbonyl; dba: Dibenzylidineacetone; EDCI: 1-ethyl-3-(3-dimethylaminopropyl)carbodiimide; HOAt: 1-hydroxy-7-azabenzotriazole; Cy: Cyclohexyl; H–G 2nd cat.: Hoveyda–Grubbs second generation catalyst.

Macrolactam **35** has been prepared using a different route, as shown in Scheme 9 [56]. An asymmetric aldol reaction between chiral oxazolidinone auxiliary-attached amide **37** and acrolein performed in the presence of TiCl_4_ was initially used to provide β-hydroxy amide **38** in good yield [57]. After four conventional steps, including cleavage of the chiral auxiliary, *t*-butyldimethylsilyl (TBS) protection, and hydrolysis, carboxylic acid **39** was obtained for the coupling reaction used to prepare diene **43**. It is noteworthy that conventional protection of the secondary alcohol in **38** using TBSCl or TBSOTf did not give satisfactory results. Therefore, cleavage of the auxiliary must be carried out prior to the TBS protection step. This detour in the route required an additional hydrolysis step.

Primary amine **42** could be also prepared simultaneously. Phenylethylamine **40** was oxidized and protected to produce aromatic iodide **41** in excellent yield. A Stille coupling reaction between **41** and vinyl stannane was performed to carry out the desired C-C bond forming reaction [58]. After spontaneous deprotection of the labile Fmoc group, the desired amine **42** was obtained. An amide coupling reaction between carboxylic acid **39** and amine **42** was carried out using EDCI and HOAt to provide diene **43** in 72% yield. The ring-closing metathesis of diene **43** was then attempted [59]. When **43** was treated with the Grubbs second generation catalyst **45** [60], the homodimer **46** was obtained in 41% yield instead of the desired macrolactam **35**. Gratifyingly, the more reactive Hoveyda–Grubbs (H–G) second generation catalyst **44 [61]** produced macrolactam **35** in 83% yield as an inseparable mixture with homodimer **46**. Because the transformation of macrolactam **35** into (+)-α-HTBZ was already reported [50], Altmann group prepared (+)-α-HTBZ following the previously reported route. The inseparable homodimer **46** was removed in the latter stages of the synthesis.

**Scheme 10:**
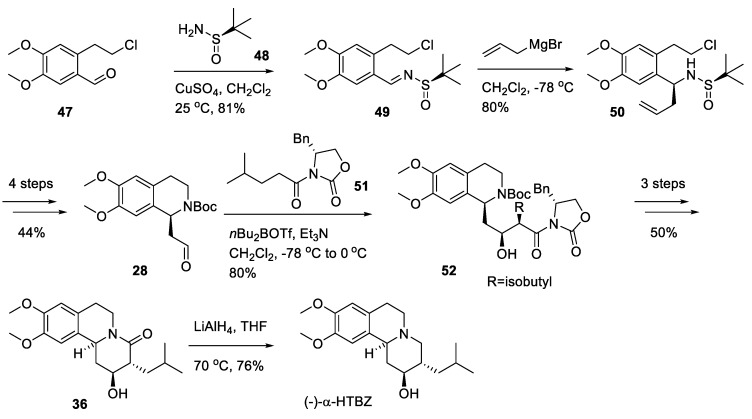
Asymmetric synthesis of (–)-α-HTBZ using a chiral sulfonamide. Bn: Benzyl.

Chiral sulfinamide **48** has also been utilized in the asymmetric synthesis of (–)-α-HTBZ, as shown in Scheme 10 [62]. Known chloroaldehyde **47** [63] was condensed with sulfinamide **48** to afford chiral imine **49** in the presence of copper sulfate [64]. The electrophilic imine **49** was then converted into homoallylic amine **50** via an allylation reaction. During this asymmetric allylation procedure, the desired product was formed as a 9:1 mixture of diastereomers. The corresponding homoallylamine **50** was transformed into known aldehyde **28,** employing a four-step procedure involving hydrolysis of the sulfinic group, nucleophilic substitution of the chloride group, Boc protection, and cleavage of the terminal alkene. Although the synthetic route from Boc-protected aldehyde **28** to α-HTBZ was already reported [19], another route was developed. An Evans-type aldol reaction between oxazolidinone **51** and the electrophilic aldehyde performed in the presence of a Lewis acid and triethylamine was used to carry out the desired C-C bond forming step to give β-hydroxy amide **52** [65]. With this key intermediate in hand, hydrolysis, Boc deprotection, and amidation steps were carried out to obtain known amide **36** in 50% yield over three steps. Amide **36** was then reduced to give (–)-α-HTBZ using a previously reported procedure [50].

### 2.4. Preparation of Radioisotopically Labeled TBZ

Radioisotopically labeled TBZ-based agents have been used to monitor the levels of neuronal VMAT2. The simple molecular structure of TBZ also assists with the development of these TBZ derivatives because it is relatively easy to quickly introduce labile radioactive isotopes into the TBZ skeleton.

**Scheme 11:**
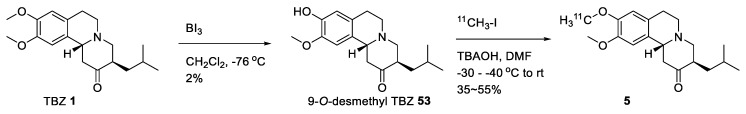
Synthesis of ^11^C-labelled-TBZ. TBAOH: Tetrabutylammonium hydroxide.

The preparation of radioisotopically labeled TBZ commenced with the synthesis of 9-demethyl TBZ **53** [31], which is suitable for rapid substitution at the phenolic OH group (Scheme 11). For 9-*O*-desmethyl TBZ **53**, direct electrophilic demethylation of TBZ **1** was carried out by the Kilbourn group. Because conventional demethylation conditions such as BBr_3_ were not successful, more reactive BI_3_ was used to produce the desired product **53** in 2% yield. Although the product yield was very low, this one-step procedure still draws the attention of medicinal chemists. With the key intermediate 9-*O*-desmethyl TBZ **53** in hand, the direct substitution of ^11^C instead of ^12^C in TBZ was initially attempted [31]. ^11^C-labelled methyl iodide was added to 9-*O*-desmethyl TBZ **53** in the presence of tetrabutylammonium hydroxide in *N*,*N*-dimethylformamide (DMF). This direct S_N_2 reaction produced a rapidly decaying TBZ derivative that simultaneously emits a positron and γ-ray irradiation. The γ-ray irradiation can be detected using PET.

**Scheme 12:**
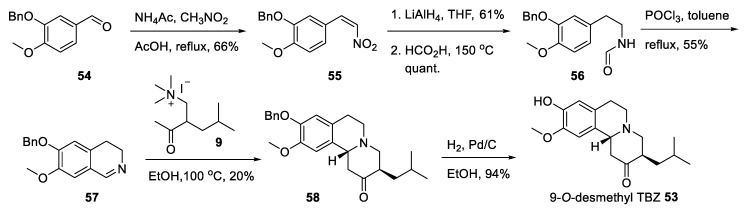
Synthesis of 9-*O*-desmethyl TBZ.

Although a short demethylation route to 9-*O*-desmethyl TBZ **53** was developed, its low chemical yield would be improved using another synthetic route. This other synthetic route is described in Scheme 12 [66]. Benzaldehyde **54** was condensed with nitromethane to afford nitroalkene **55**, which was transformed into formamide **54** via a reduction–amidation sequence [67]. A Bischler–Napieralski cyclization of **56** using POCl_3_ produced 9-benzyloxy dihydroisoquinoline **57** [68]. Classical cyclization of ammonium ion **9** and isoquinoline **57** in refluxing ethanol afforded benzyl-protected TBZ **58**. The labile benzyl protecting group was removed via hydrogenation to give 9-*O*-desmethyl TBZ **53** in 94% yield.

**Scheme 13:**
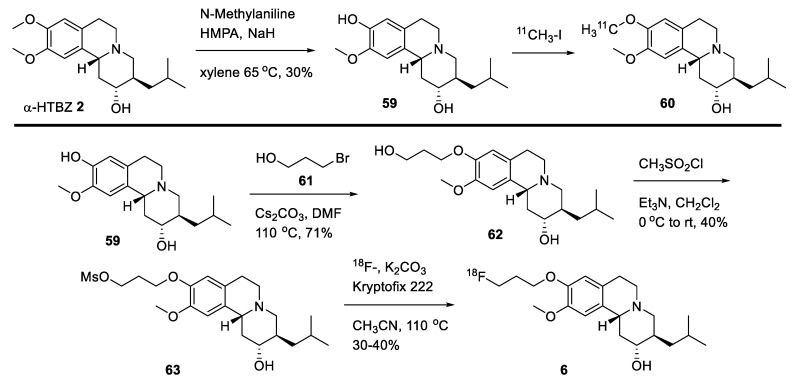
Synthesis of ^18^F-propyl-(α)-HTBZ. HMPA: Hexamethylphosphamide; Ms: Methanesulfonyl.

A radioisotopically labeled α-HTBZ derivative has also been utilized as a PET imaging agent to monitor the levels of VMAT2, as shown in Scheme 13. Selective demethylation of α-HTBZ was accomplished to afford phenol **59** in low yield [17], which was then converted to ^11^C-incorporated α-HTBZ **60** directly [69]. 9-*O*-desmethyl α-HTBZ **59** could be utilized to introduce other radionucleotides. The phenol **59** was substituted with propyloxy bromide **61** in 71% yield in warm DMF. After mesylation of the primary alcohol in **62**, an on-site generated ^18^F anion was added to obtain the desired ^18^F-propyl-α-HTBZ product **6** via a simple S_N_2 reaction [70]. Although this PET imaging agent displays similar properties to those of ^11^C-TBZ, ^18^F-propyl-α-HTBZ exhibits a longer signal duration (^18^F T_1/2_ 110 min) than ^11^C-TBZ (^11^C T_1/2_ 20 min). This prolonged duration facilitates the handling of this imaging agent.

**Scheme 14:**
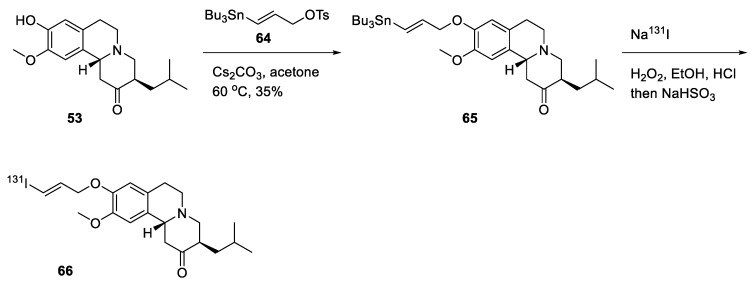
Synthesis of ^131^I-9-iodovinyl-TBZ.

Single-photon emission computed tomography (SPECT) is suitable for in vivo imaging of a target organ as well. SPECT has several advantages over PET, such as its widespread use based on inexpensive equipment and facile preparation of radioisotopes [71,72]. For this reason, ^131^I-incorporated TBZ has been prepared, as shown in Scheme 14 [72]. Unlike ^11^C or ^18^F, the introduction of ^131^I requires a significant change in the parent structure of TBZ. Although this change will affect its VMAT2 binding affinity, ^131^I is still valuable because it can be used to prepare a relatively stable in vivo imaging agent. Thus, vinyl stannane **64**, which was prepared via hydrostannylation of propargyl alcohol, was attached to 9-*O*-desmethyl TBZ **53** using an S_N_2 reaction. Iodination was carried out to obtain the desired ^131^I-9-iodovinyl-TBZ product **66** via an in situ oxidative substitution reaction. This radioactive agent was utilized to visualize rat brains.

**Scheme 15:**
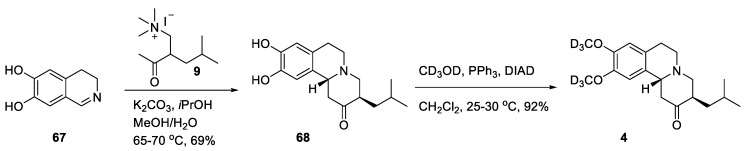
Synthesis of deutetrabenazine. DIAD: Diisopropyl azodicarboxylate.

Scheme 15 summarizes the synthesis of deutetrabenazine, a deuterium-incorporated TBZ derivative, which was the first deuterium-containing drug approved by the US FDA in 2017. Deutetrabenazine is used to treat tardive dyskinesia, an involuntary movement disorder [25]. When compared to TBZ, this deuterium-substituted derivative is superior with less-frequent and lower dosing required, which leads to less adverse effects and can be synthesized via modification of the classical TBZ synthesis. Dihydroisoquinoline **67** was cyclized to form key intermediate **68** in a single step. Finally, a Mitzunobu-type reaction between CD_3_OD and intermediate **68** was carried out in the presence of PPh_3_ and diisopropyl azodicarboxylate (DIAD). This two-step procedure provided deutetrabenazine **4** on a kilogram scale [73].

### 2.5. Preparation of a TBZ Prodrug

**Scheme 16:**
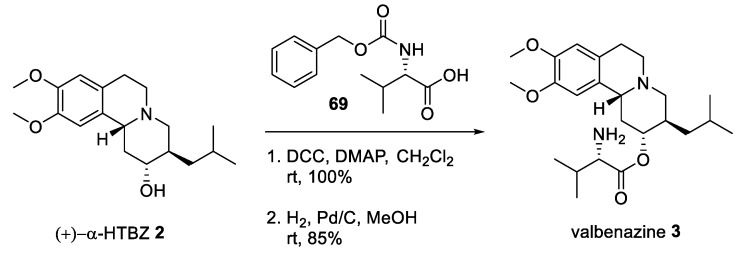
Synthesis of valbenazine. DCC: Dicyclohexyl carbodiimide; DMAP: 4-dimethylamino pyridine.

A prodrug of TBZ has been developed. Valbenazine **3** is a valine ester prodrug of (+)-α-HTBZ (Scheme 16) [74]. Standard esterification of (+)-α-HTBZ with carbamate-protected (*L*)-valine **69,** followed by hydrogenolysis of the benzyloxycarbonyl group, afforded valbenazine in a straightforward manner. Using this synthetic procedure, valbenazine proceeded to clinical trials. Valbenazine showed a prolonged duration (T_1/2_ ~20 h) when compared to TBZ (T_1/2_ ~10 h) [22,23,24]. Actually, the effective dose of valbenazine was maintained for a longer period of time; it is possible to prescribe valbenazine once daily. Valbenazine was approved for the treatment of tardive dyskinesia by the US FDA in 2017.

## 3. Conclusions

Since its first clinical use in 1971, TBZ has served as the only medication used for the treatment of chorea in HD and other related involuntary movement disorders. TBZ has improved the quality of life of a tremendous number of patients over the last 50 years. This widespread use as a medication has led to its approval by the US FDA, although TBZ still has some limitations. First, it was approved as a racemic mixture. This stereochemical issue can be solved by the development of chiral resolution or asymmetric synthesis strategies. During the establishment of an asymmetric synthesis, more efficient synthetic routes to TBZ have been studied. Secondly, pharmacokinetically, TBZ is prone to being quickly changed in vivo. It is rapidly metabolized to make it difficult to provide an effective dose inside the human body. This drawback could be overcome by development of a more stable prodrug or the incorporation of metabolism-resistant radioisotopes into the drug structure. Specifically, the latter strategy has led to the first deuterium-containing drug to be approved by the US FDA after 50 years of research in this area.

TBZ is also a valuable lead compound for the diagnosis of PD. Because there is no effective treatment regimen for PD except for *L*-DOPA/carbidopa, early diagnosis is still wanted. Although treatment with TBZ may make PD worse via the depletion of dopamine in the synaptic cleft, a trace amount of TBZ will not have a significant effect. Consequently, the use of TBZ as a radioactive tracer or ligand will be suitable for powerful imaging systems such as PET or SPECT. This type of development is still on-going, using various radionucleotides and modifications of the TBZ structure.

TBZ still has limitations in terms of its side effects and bioavailability. In addition, it does not cure the patient, but relieves the symptoms of the disease. However, advances using TBZ are still in progress. A variety of TBZ derivatives is expected.
